# SOD-YOLO: Detection Methods for Vehicles with Occlusion and Small Targets

**DOI:** 10.3390/s26206426

**Published:** 2026-10-11

**Authors:** Zongnan Liu, Yong Xu, Zepeng He

**Affiliations:** 1 School of Aerospace Engineering, Beijing Institute of Technology, Beijing 100081, China; 2 Beijing Institute of Technology Chongqing Innovation Center, Chongqing 401100, China

**Keywords:** vehicle target detection, small target detection, occluded, YOLOv13

## Abstract

UAV-based vehicle detection has important application value in the fields of intelligent transportation, urban security and emergency rescue. It is the key technology for realizing high-altitude situational awareness and dynamic monitoring. However, in this scenario, the vehicle targets generally have the problems of being small in size, densely distributed, and mutually occluded, which leads to the model being prone to feature loss, missed detections, and high false detection rates. To address these challenges, we propose a vehicle small-target and occluded-target detection method called the small object and occlusion detection method (SOD-YOLO), which is based on YOLOv13. This method includes three main innovations. First, we designed a sliding-window self-attention convolutional structure, a small object and occlusion detection convolution structure (SOD-C2f), which is based on the existing sliding-window self-attention (SWSA) structure. It is suitable for extracting features of small vehicle targets. Through the self-attention mechanism within the local window, we enhance the fine-grained expression of small target features, effectively improving the model’s perception and positioning ability for tiny vehicle targets. Afterward, we integrate the coordinate attention mechanism (CA) by explicitly modeling the interchannel dependencies and spatial position information to achieve precise semantic alignment of features at different levels, significantly enhancing the detection robustness in occluded scenarios. Finally, we employ the Space-to-Depth Convolution (SPDConv) module, which splits the spatial dimension and replaces the traditional stride convolution and pooling layer with nonstride convolution. This approach avoids the loss of fine-grained features during downsampling and further retains the key spatial information of small vehicle targets. The experimental results show that the precision, recall, average mAP@0.5 and mAP@0.5:0.95 of the proposed SOD-YOLO algorithm are 77%, 74.8%, 80.9% and 54.9%, respectively. Compared with the benchmark model YOLOv13, the three indicators increase by 3.6%, 1.1%, 3.6% and 2.6%, respectively. In conclusion, the proposed algorithm performs better in the tasks of detecting small targets and occluded vehicles and is able to better meet the precise detection requirements of vehicle targets from a high-altitude perspective.

## 1. Introduction

With the rapid development of intelligent transportation systems, urban smart security and low-altitude perception technologies, unmanned aerial vehicle (UAV) aerial photography detection, owing to its advantages of high flexibility and wide coverage, has become the core technical method for the intelligent monitoring of vehicle targets. It plays a crucial role in scenarios such as traffic flow statistics, violation behavior recognition, and road hazard investigations [1,2]. Unlike the traditional head-up monitoring perspective, the images captured by unmanned aerial vehicles from high altitudes have variable target scales and complex background environments. The vehicle targets in the picture are mostly small-scale objects and are severely affected by buildings, trees, and adjacent vehicles, which easily leads to incomplete target features and blurred boundaries, greatly increasing the difficulty of vehicle detection [3,4]. Therefore, research is being conducted on algorithms for the precise detection of small targets and occluded vehicles in this scenario. This is highly important for enhancing the intelligent perception capabilities of drones and expanding the application scenarios for low-altitude traffic monitoring [5,6].

Many object detection algorithms have been applied to vehicle detection, including traditional detection algorithms, two-stage object detection algorithms, transformer detection algorithms, and single-stage YOLO series of detection algorithms [7]. In the research of traditional vehicle detection algorithms, Yang et al. [8] combined the background subtraction method with the online Kalman filtering algorithm and proposed a vehicle detection and tracking counting method in complex traffic scenarios. This method solved the problems of efficient real-time traffic information collection, precise vehicle counting, and repeated counting in complex traffic scenarios. However, this algorithm is highly dependent on manual feature design and has poor generalization ability. When dealing with vehicle targets that are extremely small in size and have incomplete occlusions, it is prone to feature extraction failure and target omission, making it difficult to adapt to complex drone aerial photography scenarios. In the field of two-stage object detection algorithms, the R-CNN series of algorithms has been widely applied to vehicle detection tasks [9,10]. Ji et al. [11] combined R-CNN series detection technology and improved Faster R-CNN by optimizing feature extraction, multi-scale feature fusion, and essential data augmentation. They proposed a vehicle detection method for remote sensing images that solved the problem of insufficient vehicle detection accuracy in remote sensing images. However, this type of algorithm has the drawbacks of a complex network structure, a large number of parameters, and a slow inference speed and cannot meet the real-time detection requirements of unmanned aerial vehicles. Moreover, it has an insufficient ability to restore the features of occluded targets [12,13]. The detection algorithm based on transformers, leveraging its advantage of global modeling, performs exceptionally well in complex target detection tasks [14]. Krishnendhu et al. [15] combined a DETR detection model, a convolutional neural network, and a spatial pyramid pooling structure to propose the DETR-SPP single-stage vehicle detection network. This solution addresses the issues in intelligent transportation systems where real-time vehicle detection cannot simultaneously achieve speed and accuracy, is affected by occlusion in terms of detection accuracy, and lacks dedicated vehicle detection models. However, this algorithm has extremely high computational costs and training expenses, is highly demanding in terms of hardware equipment, and has the problem of weak local fine-grained feature extraction, making accurate identification of tiny vehicle targets difficult [16].

A series of specialized algorithms have recently been proposed for UAV aerial small object detection. Wei et al. [17] addressed the issue of insufficient detection accuracy for small targets in high-density scenes captured by unmanned aerial vehicles and proposed the DV-DETR algorithm. They improved the backbone, attention mechanism, and loss function of RT-DETR, taking into account both detection accuracy and real-time performance. Chen et al. [18] addressed the problem of easily lost small-target features in aerial images and proposed a semantic guided global local fusion algorithm. They utilized high-level semantics to align the low-level texture features. This effectively improved the detection accuracy of small targets by unmanned aerial vehicles. Wu et al. [19] addressed the problem of insufficient labeled samples for multi-view infrared images and poor fine-grained recognition. They proposed a multi-view infrared self-supervised representation learning recognition algorithm. Through contrastive learning for pre-training, they improved the accuracy of fine-grained recognition of infrared targets. Zhang et al. [20] addressed the issue that it is difficult to balance detection accuracy and lightweight deployment in unmanned aerial vehicle (UAV) aerial photography detection and proposed the RTUAV-YOLO lightweight model series. They designed a multi-module optimization process for extracting small target features and downsampling, which can meet the real-time inference requirements of airborne edge platforms. Wei et al. [21] addressed the problem of poor receptive field matching and weak feature extraction ability of small targets in UAV images and proposed the RFAG-YOLO receptive field attention-guided network. The receptive field guidance module enhances the fine-grained features of small targets. This effectively improves the detection accuracy of aerial small targets. Nevertheless, these approaches concentrate primarily on small target feature enhancement or infrared modal recognition and pay insufficient attention to feature aliasing and confusion induced by heavy mutual occlusion among vehicles.

The YOLO series of single-stage algorithms, with their advantages of high accuracy, low latency and a lightweight nature, have become the mainstream algorithms for real-time detection of unmanned aerial vehicles and vehicles [22,23,24]. Liu et al. [25] proposed the YOLO-DC vehicle detection framework by combining the YOLO framework, channel prior convolutional attention, and FasterNet modules. This framework addresses the challenge of real-time detection in complex environments, such as those involving changes in vehicle geometry and varying lighting and weather conditions. It enables high-precision detection of vehicles at different scales and under adverse weather conditions. Zhou et al. [26] proposed MP-YOLO, a lightweight vehicle detection algorithm. It addresses the challenges in autonomous driving of high energy consumption in AI models and the difficulty of balancing detection accuracy with deployment on edge devices. The algorithm achieves improved detection accuracy while significantly reducing the model size, thereby meeting the requirements for deployment on edge devices. As a relatively new single-stage detection algorithm, YOLOv13 relies on a lightweight backbone network, an optimized feature fusion structure and efficient detection head design, taking into account both detection accuracy and inference speed. Compared with the previous YOLO series algorithms, the YOLO algorithm has significant advantages in terms of scale adaptive detection and real-time reasoning and provides an excellent benchmark model for UAV vehicle detection tasks [27,28].

Although YOLOv13 demonstrates excellent overall performance in conventional vehicle detection tasks, it still has certain issues in regard to detecting small-scale vehicles and densely occluded vehicles in drone aerial photography scenarios. The original feature extraction module of the model has insufficient ability to capture fine-grained features of small targets, and the key features of small targets are easily lost in the downsampling process. The patching and integration method fails to effectively distinguish occluded targets from background interference, resulting in frequent false negatives and false positives in the model, making it difficult to meet the precise detection requirements for complex low-altitude traffic monitoring. To address the above issues, we propose SOD-YOLO, a small-object and occluded vehicle detection algorithm based on an improved YOLOv13. By optimizing multiple modules in a coordinated manner, we enhance the model’s detection capabilities. The core innovations are as follows:

(1) We designed the sliding-window self-attention convolutional structure SOD-C2f, which is based on the existing SWSA structure. It is suitable for extracting the features of small vehicle targets in order to address the problems of weak feature representation and insufficient feature extraction for small targets.
(2) The coordinate attention (CA) mechanism was added. It enhances the weights of effective vehicle features and suppresses interference from complex backgrounds.
(3) The SPDConv module was introduced to optimize the network’s downsampling mechanism, further improving the model’s detection accuracy for small-scale and occluded vehicles.

## 2. Proposed Vehicle Small-Object Detection Method SOD-YOLO

**Figure 1:**
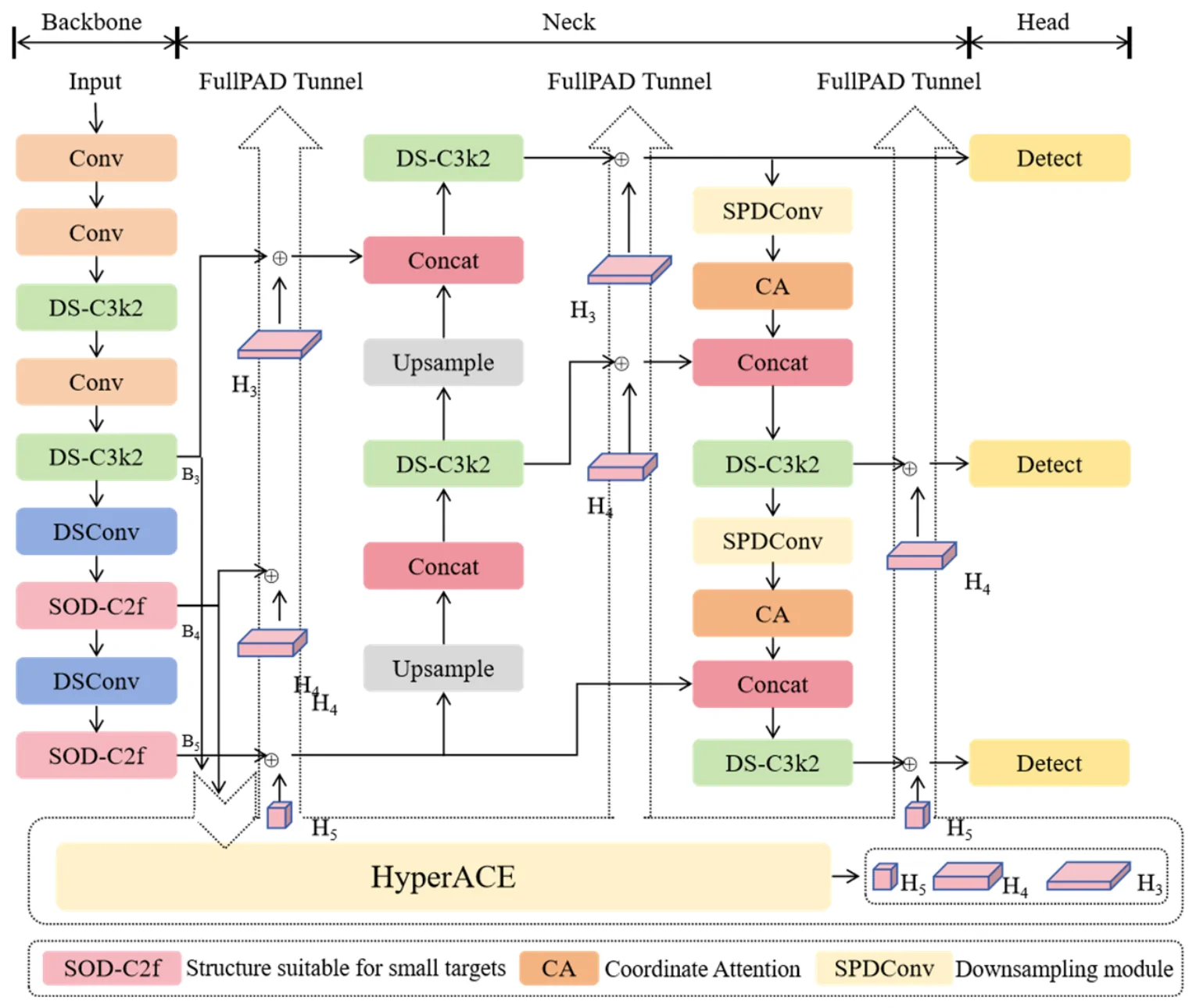
Structural diagram of the proposed SOD-YOLO model.

To address the challenges of weak small-target features and mutual occlusion of targets in vehicle detection from a drone’s aerial perspective, we propose the SOD-YOLO model for detecting small targets and occluded vehicles on the basis of YOLOv13 [29], as shown in Figure 1. In this model, we designed a sliding-window self-attention convolution structure, SOD-C2f, suitable for feature extraction of small vehicle targets. By using a local window self-attention mechanism to enhance the fine-grained feature expression of small targets, the model’s perception and localization capabilities for small vehicle targets are improved. In addition, we embedded a coordinate attention (CA) mechanism between the Conv and Concat concatenation operations to accurately model the dependency relationship between the target spatial position and channel dimension, enhance the effective feature weights of occluded vehicles, and suppress complex background interference. Moreover, we improved detection robustness in occluded scenes. Finally, in the downsampling stage of the network, we replaced the last two standard convolutional layers in the model with SPDConv modules and replaced traditional stride convolution with space-to-depth splitting to avoid the loss of key spatial features of small targets during the downsampling process and further preserve the detailed information of the vehicle target.

### 2.1. Proposed Convolutional Structure SOD-C2f Applicable to Small Targets

**Figure 2:**
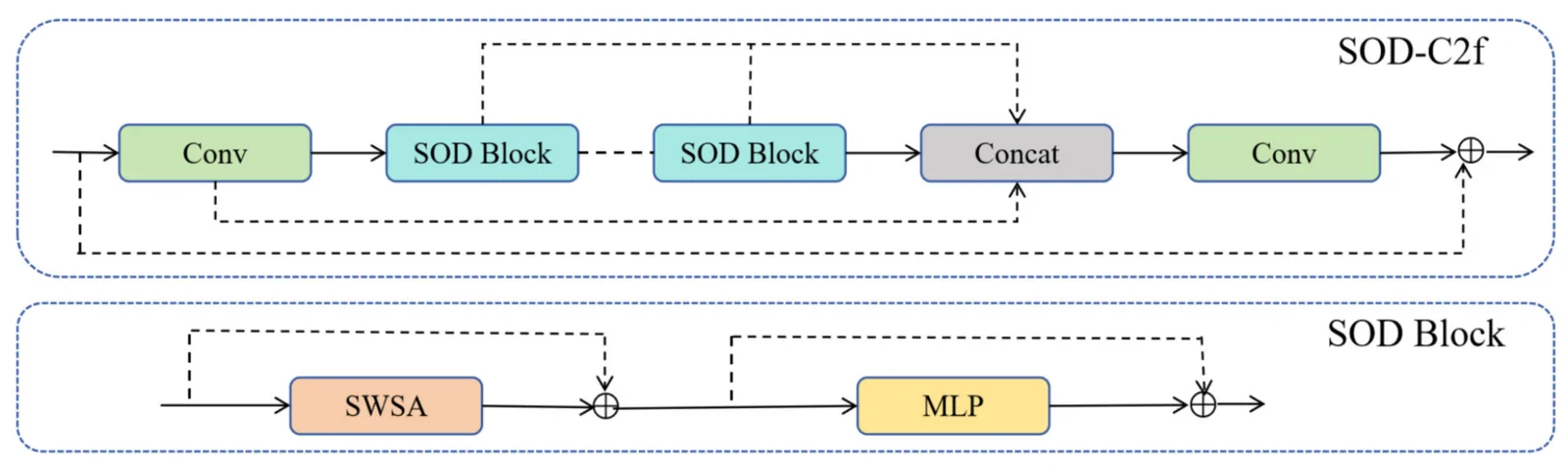
Structural diagram of the proposed SOD-C2f. SOD-C2f adopts a dual-branch architecture. The core component SOD-Block integrates DWConv and the improved PatchSA. DWConv is used for feature projection, and PatchSA is the basis for the sliding-window self-attention calculation in this paper. The main branch achieves feature enhancement by serially connecting SOD-Block units, while the residual branch retains the original feature information. Finally, feature fusion is completed by concatenation and 1 × 1 convolution.

In response to the problems of weak fine-grained features and easy loss in the downsampling and feature extraction of small vehicle targets in drone aerial scenes, we designed an improved feature extraction module, SOD-C2f, for small-target vehicle detection on the basis of the original A2C2f module of YOLOv13. Its structure is shown in Figure 2.

SOD-C2f generally adopts the lightweight dual-branch architecture of A2C2f, which consists of four components: input convolution, feature splitting, stacked attention bottleneck enhancement, and output fusion. First, the input features are compressed by a 1 × 1 convolution to complete channel compression and dimension normalization, which reduces the subsequent computational overhead while retaining the core information. Afterward, the feature is divided into two branches, and the main branch enters the feature enhancement unit composed of multiple SOD blocks in series. The other path is a direct residual branch, which is used to retain the gradient information of the original feature and prevent the gradient from disappearing.

**Figure 3:**
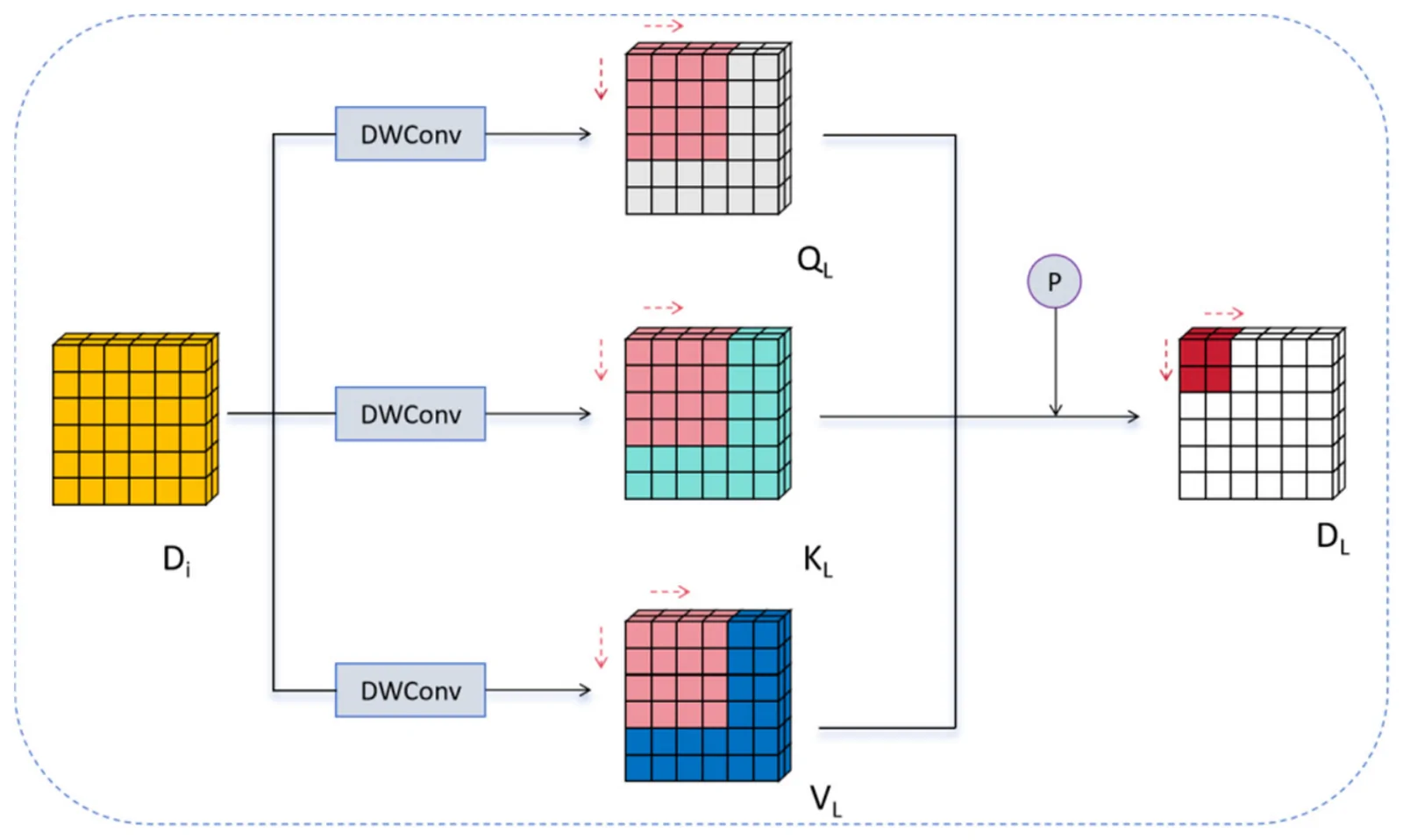
Sliding-window self-attention (SWSA).

In the core SOD-Block unit, the original attention mechanism is replaced by sliding-window self-attention (SWSA) implemented on the basis of PatchSA [30]. PatchSA is a patch-based self-attention primitive originally proposed for remote-sensing change-detection tasks, which partitions feature maps into non-overlapping local patches and executes self-attention calculation within each patch region. However, vanilla PatchSA adopts fixed non-overlapping patch division, which lacks cross-patch information interaction and is not well-suited for densely distributed tiny vehicle targets in a UAV context. Therefore, we employed a method based on sliding-window self-attention (SWSA). As shown in Figure 3, the input features are first mapped into three groups of features, query, key, and value, through depth-wise separable convolution (DWConv). Self-attention is subsequently calculated within a fixed-sized local sliding window, focusing only on the spatial dependencies among neighboring pixels within the window. This approach not only avoids the high computational cost of global attention but also precisely captures the local detailed features of small vehicle targets. Moreover, a relative-position encoding mechanism is introduced. Modeling the spatial positional relationships of features within a window further enhances the spatial positional identification of small targets. This effectively alleviates the problem of confusion between small target features and background clutter. On this basis, the SOD-Block also achieves feature enhancement through an MLP feedforward network and residual connections. Without significantly increasing the model’s parameter quantity, it further improves the feature representation ability.

To quantitatively describe the operation process of the aforementioned sliding-window self-attention mechanism, relative-position encoding is introduced, and its calculation expression is shown in Equation (1).

(1) $$\mathrm{SWSA}(Q,K,V)=\mathrm{Softmax}\left(\frac{QK^{T}}{\sqrt{d_{k}}}+R\right)V$$

In this formula, $Q,K,V\in R^{H\times W\times d_{k}}$ represent the feature projections obtained by the depth-wise separable convolution DWConv. *R* is the relative-position encoding matrix. *D_k_* is the dimension of the single-head attention feature.

In terms of tensor dimensions, the input feature tensor **X**_in_ ∈ ℝ*^H^*^×*W*×*C*^; after the sliding-window self-attention operation, the output feature **X**_swsa_ ∈ ℝ*^H^*^×*W*×*C*^, with the spatial resolution and the number of channels remaining unchanged.

Finally, the enhanced features of the main branch are concatenated with the directly connected residual branch and fused with a 1 × 1 convolution for output. The feature extraction process of the entire SOD-C2f module is complete. After the internal feature enhancement is completed in SOD-Block, the enhanced features of the main branch and the residual branch are concatenated and fused. This fusion process is defined by Equation (2).

*X*_out_ = Conv_1×1_(Concat(*X*_swsa_,*X*_res_)) (2)

In this formula, **X**_swsa_ represents the enhanced feature output by the series SOD-Block. **X**_res_ denotes the features of the residual shortcut branch.

This structure focuses on key areas of small targets through local-window self-attention and enhances spatial positioning capability with relative-position encoding. On the premise of ensuring model lightweighting and inference efficiency, the feature extraction accuracy and anti-interference ability of small vehicle targets are effectively improved, relieving the problem of insufficient characterization of small target features and loss of detail in traditional convolution. This provides more discriminative feature inputs for subsequent feature fusion and detection heads.

### 2.2. Added Coordinate Attention Mechanism

**Figure 4:**
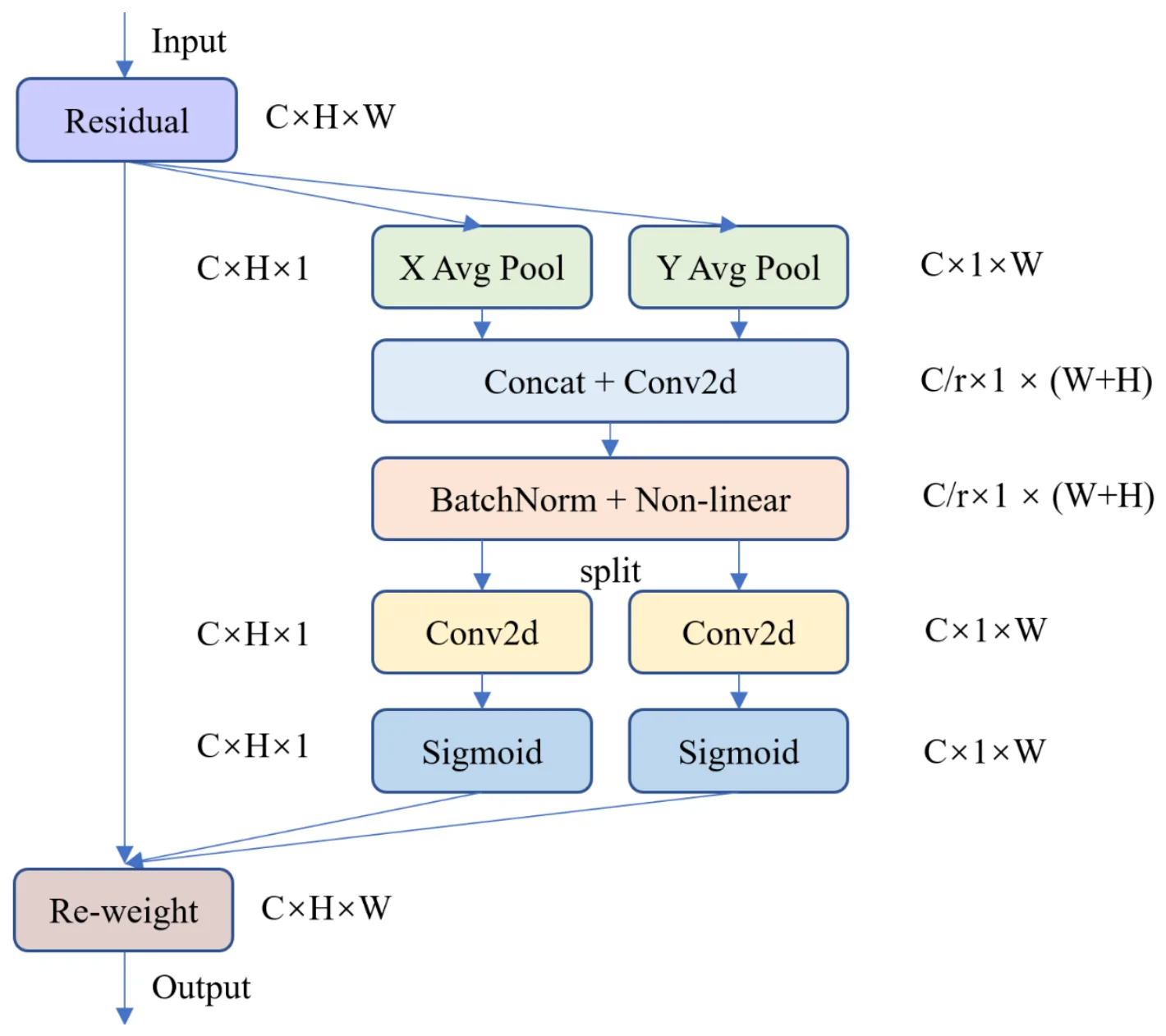
Structural diagram of the CA attention mechanism [32].

To address issues such as vehicles obscuring one another and features being confused with complex backgrounds in drone aerial photography scenarios, we incorporated a coordinate attention (CA) mechanism [31] into the feature fusion stage of the neck network. In our SOD-YOLO model, two CA modules are embedded in total. They are separately deployed after the two SPDConv downsampling modules and before each Concat feature-splicing operation, as shown in Figure 1 and Figure 4. It is embedded between the Conv layer and the Concat operation to jointly model the spatial position and channel dimension of the features, further enhancing the detection robustness of occluded vehicles and small targets. It is worth noting that coordinate attention is essentially a lightweight attention unit. It employs one-dimensional global-average pooling and low-dimensional convolutional transformation. Therefore, the additional parameters and computational complexity introduced are relatively small. Unlike the high-weight global self-attention, CA does not cause a sharp increase in model complexity.

Unlike traditional channel attention, which focuses only on the weight differences between channels, CA explicitly models the positional information in both the horizontal direction and the vertical direction. By encoding global spatial information into the channel attention mechanism, the model can accurately capture the spatial positions and occlusion boundary features of small vehicle targets. Specifically, coordinate attention first encodes the feature map along the horizontal and vertical directions through two one-dimensional global-average pooling operations, generating feature vectors containing position information. Afterward, through 1 × 1 convolution and nonlinear activation functions, it learns the dependency between channels and spatial dimensions, generating attention weights with position perception. Finally, this weight is weighted with the original feature map to enhance the effective features of occluded vehicles and small targets and suppress interference from background noise and irrelevant areas.

After the bidirectional pooling operation is completed, attention weights with spatial perception capabilities are generated through convolution and activation, and the original features are weighted and calibrated. The mathematical expression of this process is shown in Equations (3)–(5).

*A_H_* = Sigmoid(Conv_1×1_(Pool*_H_*(*X*))) (3)

*A_W_* = Sigmoid(Conv_1×1_(Pool*_W_*(*X*))) (4)

*X*_ca_ = *X*⊙*A_H_*⊙*A_W_* (5)

Here, Pool*_H_* and Pool*_W_* respectively represent one-dimensional global average pooling along height and width. ⊙ denotes an element-wise multiplication operation.

In terms of tensor dimensions, the input feature **X** ∈ ℝ*^H^*^×*W*×*C*^ undergoes CA calibration to produce the output feature **X**_ca_ ∈ ℝ*^H^*^×*W*×*C*^. The tensor dimensions do not change before and after the processing.

In the SOD-YOLO model, the CA attention mechanism is deployed between the Conv layer and the Concat layer, enabling weighted calibration of high- and low-level features before feature fusion. On the one hand, it enhances the positional information of shallow fine-grained features, preventing the small target vehicle features from being submerged during the fusion process. On the other hand, it imposes spatial constraints on high-level semantic features, improving the discrimination between occluded vehicles and the background. This helps alleviate the problems of missed detection and false detection in occluded scenarios. By introducing the CA attention mechanism, the model can more accurately focus on the vehicle target area. Specifically, in dense occlusion and complex background scenarios, it effectively improves the feature recognition and detection stability of small targets and occluded vehicles.

To further analyze the computational overhead brought by the aforementioned improved module, the theoretical computational complexities of each core sub-module are presented below. The theoretical computational complexity of SWSA limited to operations within a local window is *O*(*HWC*^2^ + *H*^2^*W*^2^*C*). The computational overhead of the coordinate attention module is relatively low, with a complexity of O(HWC). Therefore, the overall computational load of SOD-C2f mainly originates from the sliding-window self-attention part.

### 2.3. SPDConv Downsampling Module

In the original YOLOv13 network, the backbone and neck networks achieve multilevel downsampling through standard convolution with a stride of 2. This type of downsampling method discards spatial pixels at fixed intervals. For vehicle targets with small scales and extremely low pixel proportions in drone aerial images, it is very likely that the vehicle contours, corners, and other fine-grained discriminative features are lost. Especially in scenarios with dense vehicle occlusion, after multiple layers of stride convolution compression, the effective features of small targets and occluded vehicles significantly decrease. This directly leads to problems such as missed detections and positioning deviations. To address the irreversible loss of spatial information caused by traditional stride convolution, we introduce the SPDConv (Space-to-Depth Convolution) lossless downsampling module. We replace all the standard Conv layers at the tail of the YOLOv13 network that performs the downsampling task with this SPDConv module. From the bottom layer, we retain the complete spatial features of small-scale and occluded vehicles.

**Figure 5:**
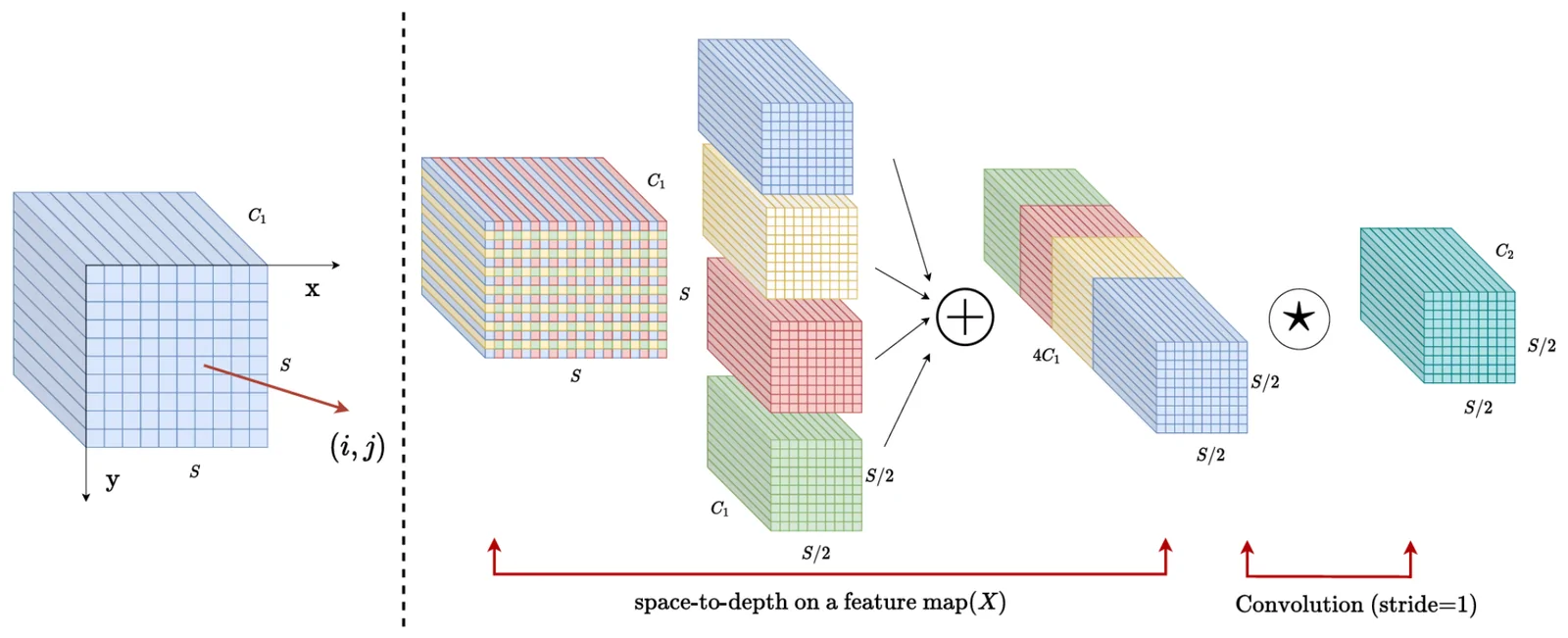
The relevant structure of SPD-Conv.

SPDConv is composed of a spatial depth transformation layer and a standard convolution without stride and is connected in series [33]. The overall structure is shown in Figure 5. Unlike the downsampling logic of stride convolution, which directly discards pixels, the SPD layer employs a parameter-free tensor rearrangement operation. With a scaling factor of 2, each 2 × 2 spatial neighborhood within the input feature map $X\in {\mathbb{R}}^{S\times S\times C_{i}}$ is split and reorganized to generate four sub-feature maps and concatenated along the channel dimension. The mathematical expression is as follows:

*f_x_*_,*y*_ = *X*[*x*:*S*:*scale*, *y*:*S*:*scale*], *x*,*y* ∈ {0,1} (6)

After stitching, a new feature map $X^{\prime }\in {\mathbb{R}}^{\frac{S}{2}\times \frac{S}{2}\times 4C_{i}}$ is obtained, achieving a halving of the feature map size and synchronous expansion of the channel dimension. Throughout the process, no pixel loss occurs, and the edges, textures, and other detailed information of the aerial vehicle are completely retained. After the spatial rearrangement is complete, it is connected to a regular convolution with a step size of 1 to fuse and compress the expanded channel features. The final downsampled feature $X^{\prime \prime }\in {\mathbb{R}}^{\frac{S}{2}\times \frac{S}{2}\times C_{2}}$ is obtained, which not only compresses the feature map size but also uses learnable convolution to filter the channel dimension information without introducing additional information loss.

In the SOD-YOLO model, SPDConv precisely replaces the last two layers of the stride-2 convolution in the network. At this position, the output feature map has a lower resolution, and the original stride convolution severely damages the features of small vehicles. SPDConv can completely retain the weak pixel features of vehicles during the downsampling stage, avoiding the situation where small targets are covered by background noise in the deep features. Moreover, SPDConv can utilize a lossless rearrangement mechanism to retain the gradient information of occlusion boundaries, alleviating the false detection problem caused by the blurring of features in occluded areas.

## 3. Experiments and Results

### 3.1. Experimental Data

We selected the DroneVehicle drone aerial vehicle detection dataset as the experimental basis, which covers real low-altitude scenes such as cities and suburbs and has problems such as a high proportion of small targets and dense vehicle occlusion. However, the original data contain some invalid empty frames without vehicle targets. If the original dataset is directly used for model training, invalid samples will continue to use model training resources and interfere with feature learning. Therefore, we selected the dataset after preprocessing [34]. After preprocessing, the final dataset consisted of 9908 image samples. Among these, there were 8442 training set images, 106,115 annotated vehicle targets, 1466 test set images and 23,205 annotated vehicle targets, effectively eliminating interference from invalid data and enhancing the feature recognition of vehicle targets in effective samples. High-quality and targeted data support for the training and testing of the SOD-YOLO algorithm will be provided in the future.

### 3.2. Experimental Configuration

The proposed SOD-YOLO algorithm follows the loss function framework of YOLOv13. The optimization objective is composed of classification loss, localization loss, and distribution focus loss, ensuring a fair comparison with the benchmark model. In addition, all the experiments were conducted on Ubuntu operating system servers, and the model improvement, training and inference processes were implemented on the basis of the PyTorch 2.4.1 deep learning framework.

In terms of training hyperparameters, all models adopt the identical training hyper-parameters as the original YOLOv13. The input image resolution is 640 × 640 pixels, and the batch size is set to 16. Exponential Moving Average (EMA) is enabled during training for weight smoothing. We employ the SGD optimizer with an initial learning rate set to 1 × 10^−2^; the weight decay coefficient is set to 5 × 10^−4^; and the optimizer momentum is 0.937. The learning rate scheduler adopts the cosine annealing strategy, and the total number of training epochs is 100 to ensure that the model fully converges. The data augmentation pipeline is consistent with native YOLOv13, including random horizontal flip, mosaic augmentation, mix-up augmentation, random scaling, and random color jitter. The mosaic probability is set to 0.5 and the mix-up probability is set to 0.1.

For test-time inference, the input resolution remains 640 × 640, consistent with YOLOv13 original settings. The confidence threshold is set to 0.25, and the Non-Maximum Suppression (NMS) IoU threshold is set to 0.7. These inference thresholds are fixed for the baseline, all ablation subgroups, and comparative models.

### 3.3. Evaluation Indicators

To comprehensively and objectively evaluate the performance of vehicle detection algorithms in unmanned aerial vehicle (UAV) aerial photography scenarios, we selected precision, recall, average precision (AP), and mean average precision (mAP) as the core evaluation indicators. The range of values for each indicator is [0, 1], with higher values indicating better model performance.

Precision is used to evaluate the model’s prediction results, representing the probability that the actual outcome is a vehicle target among all the results predicted as vehicle targets. It is used to measure the accuracy of the model’s detection, and its expression is as follows:

(7) $$\mathrm{precision}=\frac{\mathrm{TP}}{\mathrm{TP}+\mathrm{FP}}$$

The recall rate is evaluated on the basis of the original samples, representing the probability that all samples actually belonging to the vehicle target are correctly detected by the model. It is used to measure the model’s ability to cover the identification of vehicle targets, and its expression is as follows:

(8) $$\mathrm{recall}=\frac{\mathrm{TP}}{\mathrm{TP}+\mathrm{FN}}$$

The average accuracy is used to measure the detection performance of a model for a single vehicle category, which is equivalent to the area under the precision–recall (PR) curve. The higher the AP value is, the higher the detection accuracy of the model for that category of target. Its expression is as follows:

(9) $$\mathrm{AP}=\int_{0}^{1}P(R)\mathrm{dR}$$

where P(R) represents the precision rate corresponding to different recall rates R.

mAP is the core evaluation metric in the field of object detection and is used to measure the comprehensive detection performance of a model across all categories. We focus on the following two key indicators:

(1) mAP@0.5: The average value of the AP for all categories when the intersection over union (IoU) threshold is set to 0.5. This focuses on evaluating the model’s overall recognition ability for vehicle targets.
(2) mAP@0.5:0.95: The average value of the AP for all categories when the IoU threshold is 0.5–0.95 with a step size of 0.05. This comprehensively assesses the model’s localization accuracy and recognition accuracy.

The general expression of mAP is as follows:

(10) $$\mathrm{mAP}=\frac{\sum_{i=1}^{C}AP_{i}}{C}$$

where *AP_i_* represents the average precision of the *i*-th type of target and C represents the total number of categories. Additionally, the intersection over union (IoU) is used to measure the overlap between the predicted box and real box, which serves as the basis for calculating the aforementioned indicators. It is defined as follows:

(11) $$\mathrm{IoU}=\frac{\mathrm{Area}({\mathrm{Box}}_{\mathrm{pred}}\cap {\mathrm{Box}}_{\mathrm{gt}})}{\mathrm{Area}({\mathrm{Box}}_{\mathrm{pred}}\cup {\mathrm{Box}}_{\mathrm{gt}})}$$

where *Box_pred_* represents the bounding box predicted by the model and *Box_gt_* represents the actual annotated bounding box.

### 3.4. Baseline Comparison

This study aims to develop a target detection model with higher accuracy based on YOLOv13. To verify that the performance improvement is due to structural optimization rather than random training, we set five random seeds and used the same hyperparameters to train YOLOv13 and SOD-YOLO, constructing paired experiments. As shown in Table 1, the indicators are presented as mean ± standard deviation. The results indicate that SOD-YOLO achieved a significant improvement in detection performance on the validation set compared to YOLOv13.

### 3.5. Ablation Experiment

**Figure 6:**
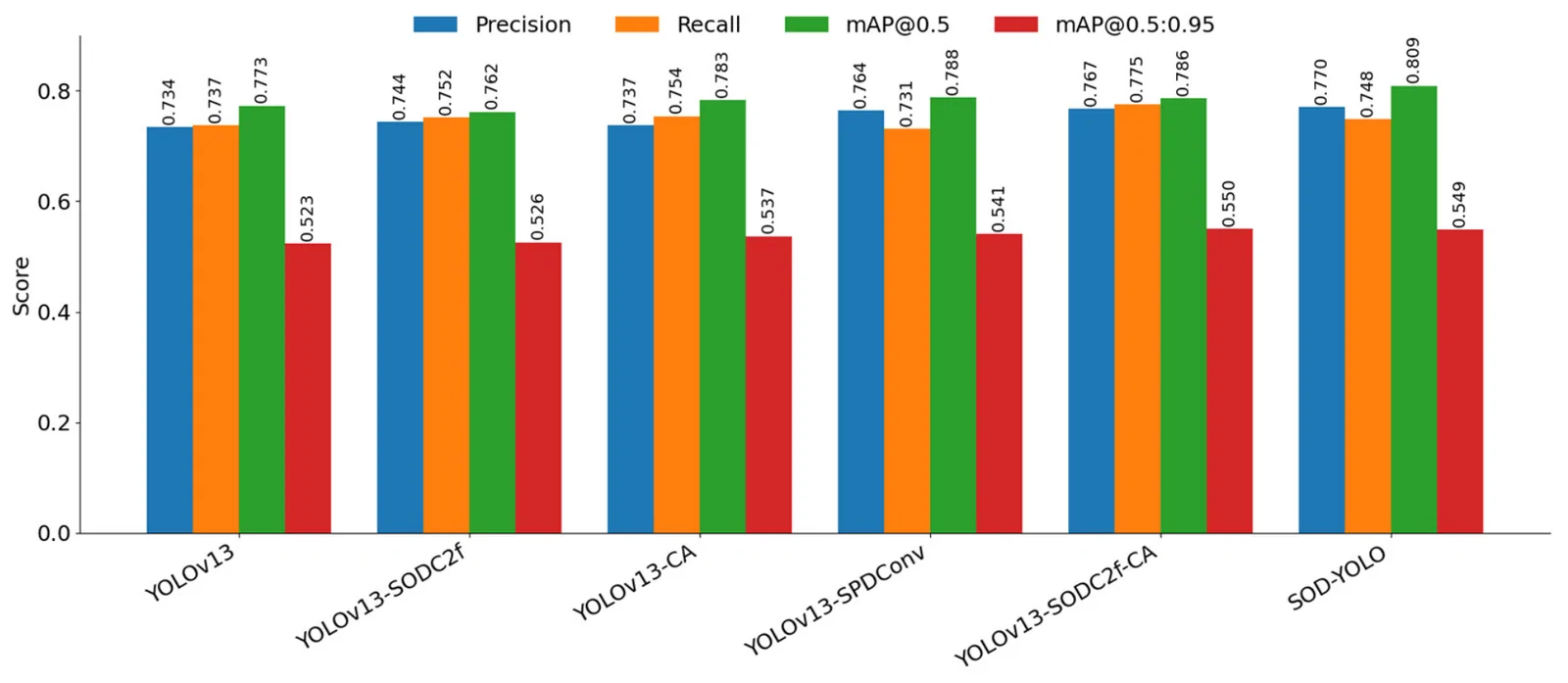
Graph showing the results of different indicators of the ablation experiment.

To verify the effectiveness of the SOD-C2f feature extraction module, the CA coordinate attention mechanism, and the SPDConv lossless downsampling module in the unmanned aerial vehicle (UAV) aerial vehicle detection task, we used YOLOv13 as the baseline model and designed six sets of ablation comparison experiments. We tested the performance gains of individual modules, combined modules, and the complete improvement scheme. The experimental results and visualizations are shown in Table 2. To more clearly display the contribution of each module to different indicators, we constructed an ablation experiment bar chart, as shown in Figure 6.

From the performance of the baseline model YOLOv13, its precision is 0.734, the recall is 0.737, the mAP@0.5 is 0.773, and the mAP@0.5:0.95 is 0.523. These figures represent the basic performance of the current single-stage detection algorithm in the unmanned aerial vehicle scenario. On this basis, after each improvement module is introduced one by one, all the indicators show varying degrees of improvement. After the SOD-C2f module alone is added, the precision and recall of the model increase to 0.744 and 0.752, respectively, and the mAP@0.5:0.95 slightly improves to 0.526. This proves that this module can effectively enhance the fine-grained feature extraction ability of small vehicle targets. After the CA attention mechanism alone is embedded, the recall increases to 0.754, and the mAP@0.5 and mAP@0.5:0.95 increase to 0.783 and 0.537, respectively. This demonstrates the advantages of coordinate attention in modeling spatial position information and alleviating confusion between occluded targets and the background. After replacement with the SPDConv module alone, the precision increases to 0.764, and the mAP@0.5 and mAP@0.5:0.95 increase to 0.788 and 0.541, respectively. This finding verifies the key role of lossless downsampling in preserving the key details of small target features.

Further tests were conducted to evaluate the collaborative effect of the multi-module combination. From the results in Table 2, it can be seen that when the baseline YOLOv13 only incorporates the SPDConv module, the precision is improved, but the recall rate drops from 0.737 to 0.731. The SPDConv retains fine-grained spatial information through lossless downsampling, thereby enhancing the precision. However, it also introduces additional background high-frequency noise, which interferes with the detection of some difficult occlusion samples, resulting in a decrease in recall capability. When the SPDConv works together with the SOD-C2f and CA attention module, this recall loss can be compensated. Additionally, compared to the SOD-C2f + CA combination, the mAP@0.5:0.95 value after combining all modules drops slightly to 0.549. The mAP@0.5:0.95 has a high requirement for bounding box localization accuracy. The additional feature noise brought by SPDConv will slightly interfere with the regression of the boxes under strict IoU conditions. Even with this slight drop, the complete model achieves the optimal mAP@0.5 and has the best overall performance in the drone vehicle detection task.

In addition, we also explored the effect of sliding window size on SWSA through ablation experiments, as shown in Table 3. When the window size is set to 4, the receptive field is insufficient to capture contextual information of adjacent occluded vehicles, resulting in relatively low detection accuracy. When the window size increases to 8, overlapping sliding windows can effectively cover the entire vehicle target and capture the contextual dependencies between dense vehicles, thereby achieving optimal detection performance. Further increasing the window size to 16 resulted in a certain decrease in the performance of the model. Therefore, choosing a window size of 8 can enable the model to achieve better performance.

To better explore the key hyperparameters of the SOD-YOLO model, the deployment positions of components, and the impact of internal components of the modules, we conducted a series of supplementary ablation experiments. The summary of the experimental results is presented in Table 4, Table 5, Table 6 and Table 7. For the multi-head self-attention within SWSA, we set the number of attention heads to 2, 4, and 8 for comparison. Too small a number of attention heads will limit the ability of feature subspace partitioning, while too large a number will introduce computational redundancy and fail to bring significant performance improvement. The four attention heads achieved the optimal comprehensive performance. We further explored the two deployment orders of SPDConv compared to CA. Placing SPDConv before CA can reserve more fine-grained features for the subsequent spatial-channel calibration, resulting in better detection indicators. For the CA module, we compared two insertion positions before and after feature concatenation. The experiment proved that completing feature calibration before multi-scale feature fusion can effectively suppress background interference and enhance the features of occluded targets. We also verified the necessity of relative-position encoding in SWSA. Removing the relative-position encoding causes the model to lose the ability to perceive spatial offsets within the sliding window, resulting in a significant decline in the detection performance for dense, occluded vehicles. The above ablation results fully demonstrate the rationality of the hyperparameter settings and module layout scheme we ultimately selected.

**Figure 7:**
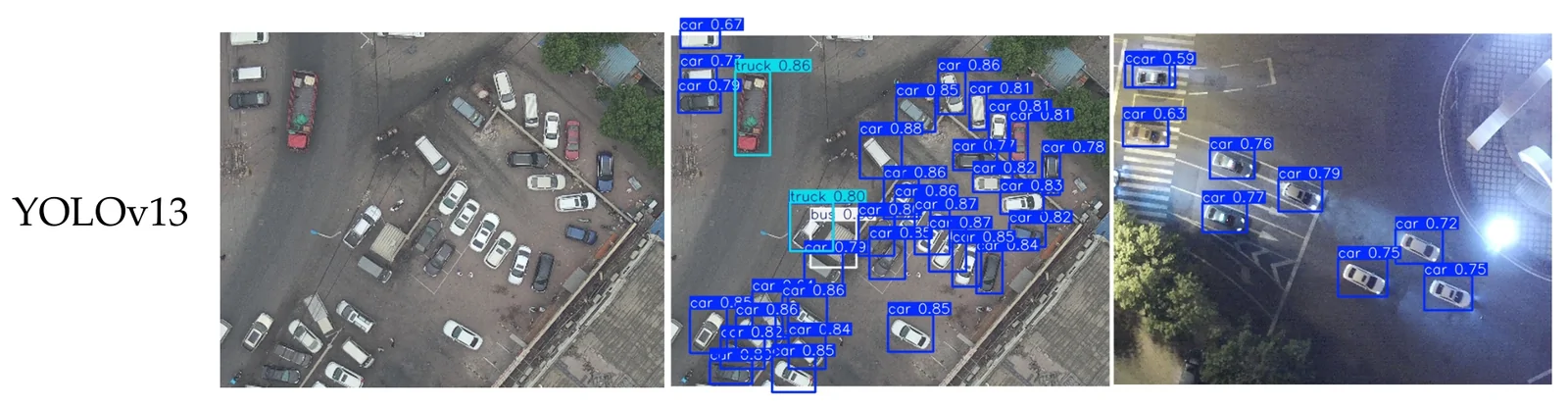

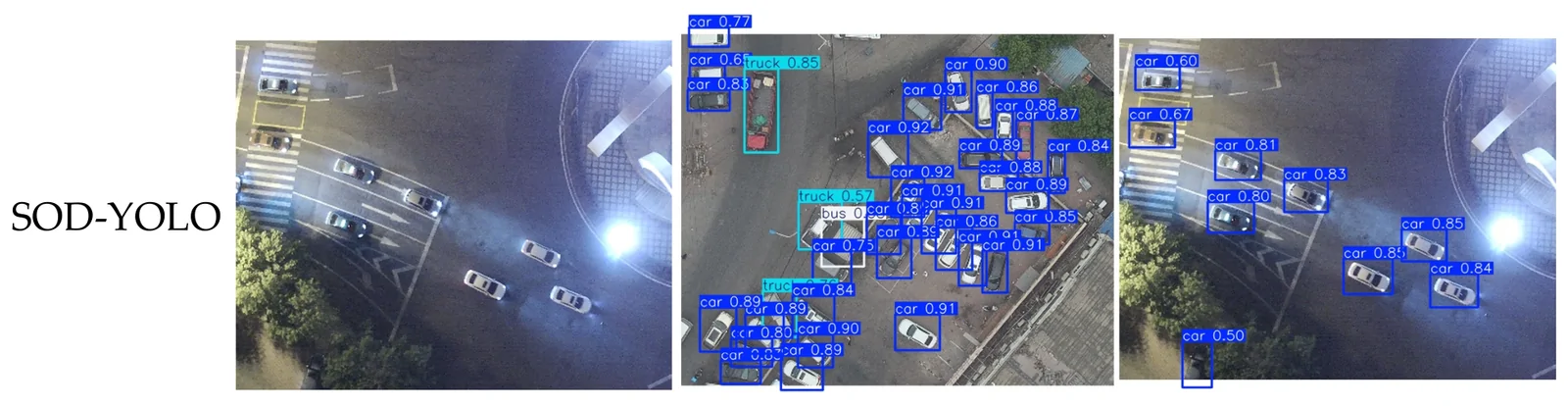
Qualitative detection comparison results between baseline model and SOD-YOLO on DroneVehicle. Representative aerial images with dense vehicles and complex backgrounds are selected for comparison. The left column is the prediction result of the baseline model, and the right column corresponds to the prediction result of the proposed SOD-YOLO. The comparison intuitively demonstrates that SOD-YOLO reduces missed detections for small-sized and mutually occluded vehicle targets.

Compared with YOLOv13, SOD-YOLO can more completely recognize vehicles with dense occlusions and small-scale vehicles, significantly reducing the number of missed detections and false detections and increasing the confidence score, as shown in Figure 7. It shows the qualitative detection comparison visualization results between the baseline YOLOv13 model and our proposed SOD-YOLO on the DroneVehicle dataset. The selected images cover multiple representative UAV aerial scenarios, including dense traffic, complex background interference and heavily occluded vehicle targets. It can be intuitively observed that the baseline model easily generates missed detections for tiny vehicles and heavily occluded vehicles. By contrast, SOD-YOLO can detect most of these hard samples, which verifies the effectiveness of our improved modules for occluded and small-sized vehicle detection. SPDConv has the most significant improvement in precision and mAP@0.5:0.95; CA attention performs best in terms of recall; and SOD-C2f provides the basis for fine-grained feature enhancement. After the collaboration of these three, the overall performance is maximized.

The results of the ablation experiments clearly demonstrate that the three improved modules we propose can enhance the performance of unmanned aerial vehicle (UAV) aerial vehicle detection in different dimensions. Compared with the baseline YOLOv13, the SOD-YOLO model achieved increases of 3.6% in terms of mAP@0.5 and 2.6% in terms of mAP@0.5:0.95. It effectively solved problems such as the loss of small-target features and the missed or incorrect detection of occluded targets. This provides a better solution for precise vehicle detection in complex low-altitude scenarios.

**Figure 8:**
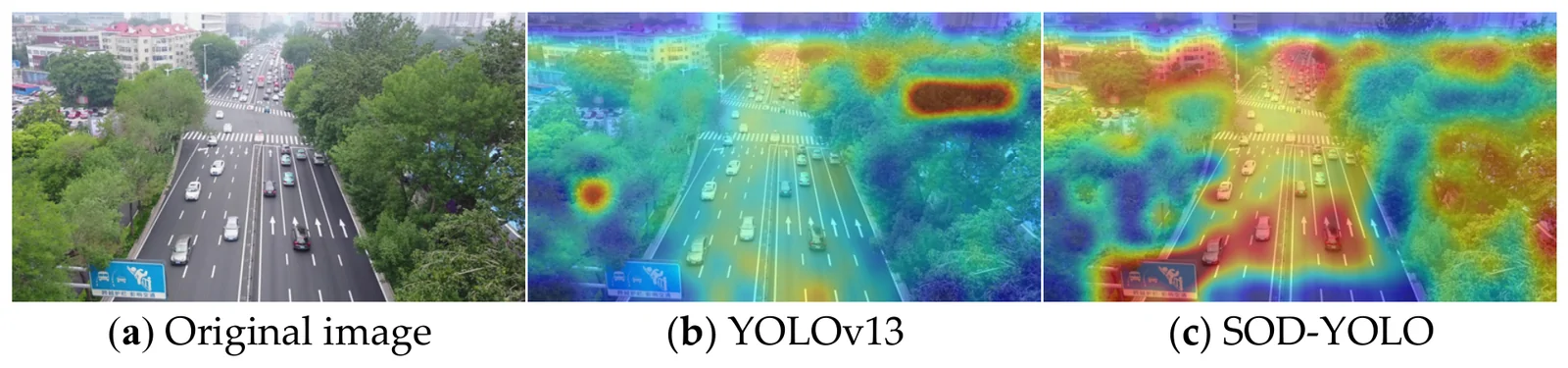
Comparison of heat maps before and after improvement.

**Figure 9:**
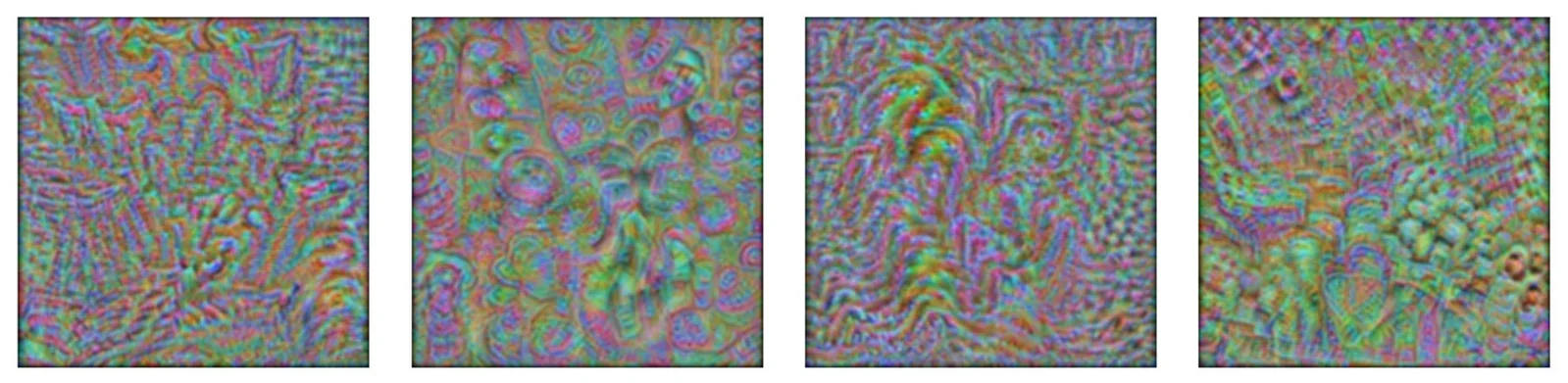

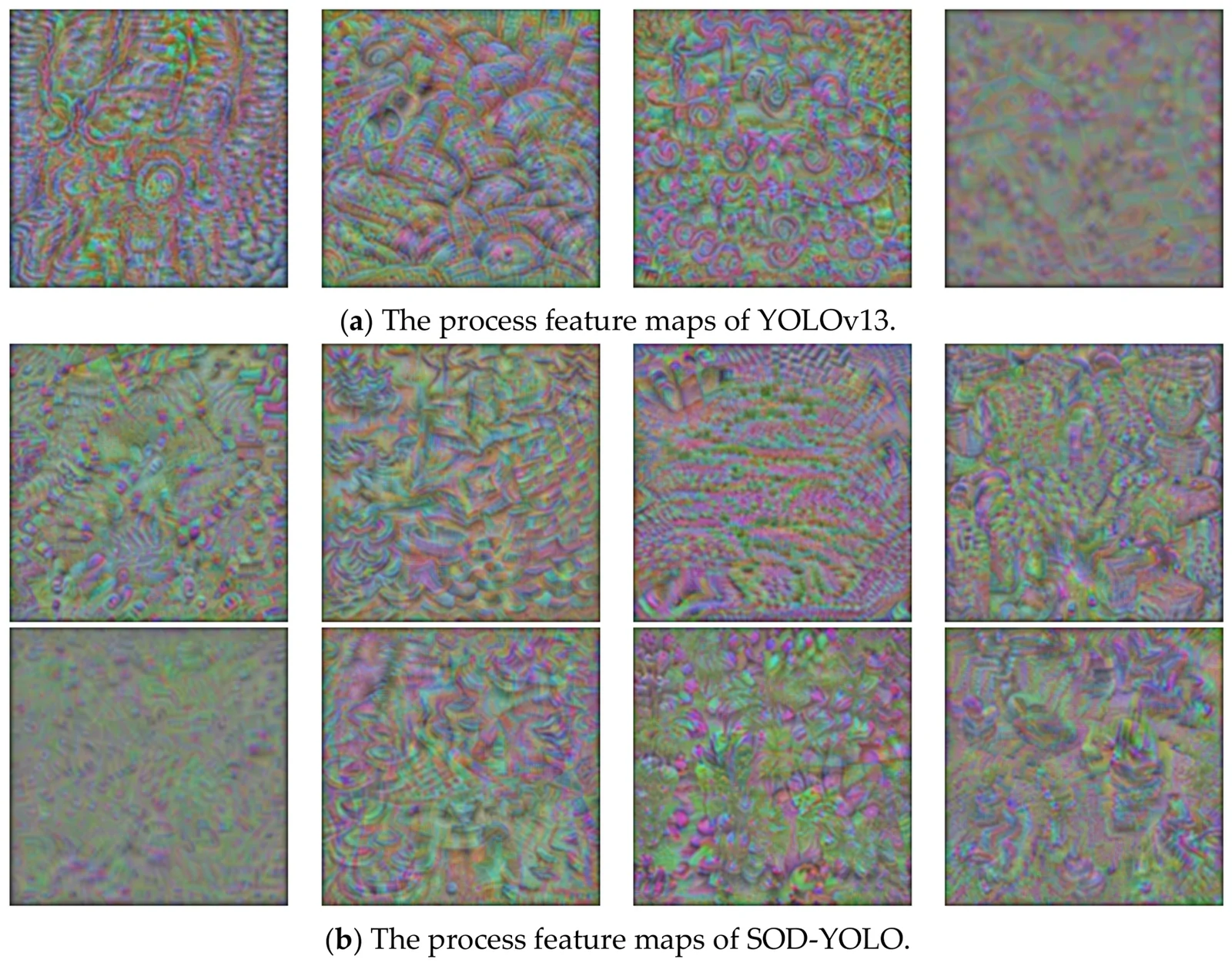
Comparison of feature maps at the same position before and after improvement.

To verify the issue of fine-grained feature loss in the original YOLOv13 when dealing with small targets and occluded vehicles from the drone’s perspective, we conducted a feature-level comparative visualization analysis. The results are shown in Figure 8 and Figure 9. We selected test set samples and presented three types of visualization results: intermediate-layer feature maps, Grad-CAM heat maps, and activation response maps. From the comparison of the intermediate feature maps, it can be seen that the original YOLOv13 has scattered and incomplete feature responses in the areas of tiny and occluded vehicles. Some vehicle contour textures are blurred or even disappear, indicating the loss of fine-grained features during the downsampling and feature extraction process. While SOD-YOLO can better retain the texture and contour features related to vehicles, the Grad-CAM heat map indicates that the attention response of the original YOLOv13 is relatively scattered. Some heat weight spreads to the road, vegetation, and other background areas. The response to occluded and small-sized vehicle bodies is weak. In contrast, the heat map of SOD-YOLO is relatively concentrated and can cover the vehicle target area. The above feature-level visualization analysis provides intuitive evidence. It confirms that the original YOLOv13 does indeed have fine-grained feature loss in the task of drone aerial photography of small targets and mutually occluded vehicles. The improvement in this paper effectively alleviates this problem.

### 3.6. Comparison with Other Advanced Detection Algorithms

**Figure 10:**
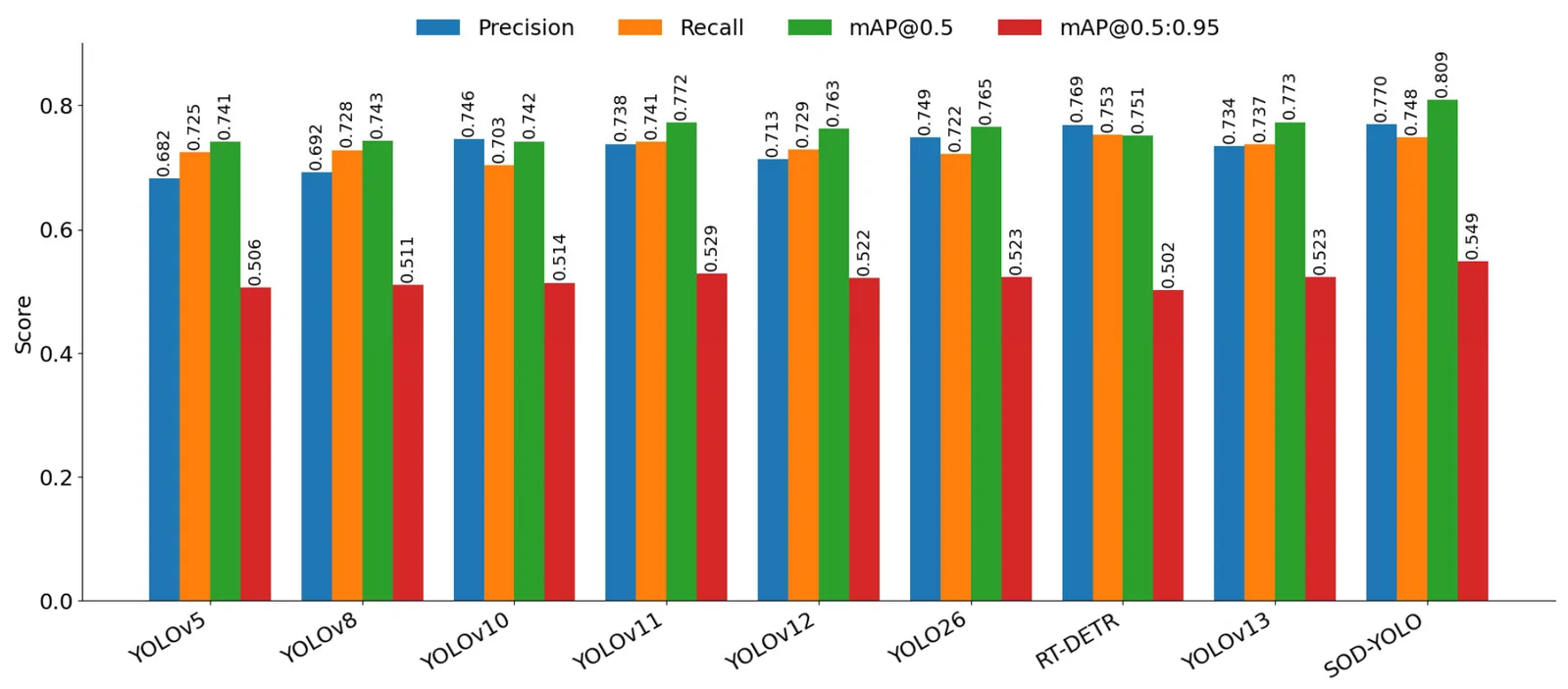
Bar chart presenting the results of the ablation experiment.

To further verify the comprehensive advantages of our proposed SOD-YOLO algorithm in the unmanned aerial vehicle (UAV) aerial vehicle detection task, we conducted a comparative experiment by selecting the mainstream detection algorithms YOLOv5 [35], YOLOv8 [36], YOLOv10 [37], YOLOv11 [38], YOLOv12 [39], YOLOv26 [40], YOLOv13 [29], and the detection algorithm based on Transformer RT-DETR [41]. We evaluated the performance differences of each model under the same dataset, training configuration, and testing environment. The results are shown in Table 8. Some of the algorithm data for the comparative experiments were derived from published results [34]. To facilitate the observation of the performance differences among the algorithms, we have drawn a bar chart, as shown in Figure 10.

With respect to the quantitative indicators, the proposed SOD-YOLO algorithm performs optimally in terms of all the core evaluation metrics. The precision reaches 0.770, which is 3.6% higher than that of the baseline YOLOv13. The recall is 0.748, which is second only to that of RT-DETR (0.753) and is 1.1% higher than that of YOLOv13, demonstrating a lower false negative rate. The mAP@0.5 is 0.809, which is 3.6% higher than the optimal baseline YOLOv13 and 5.8% higher than RT-DETR. It is the best among all the compared algorithms. The mAP@0.5:0.95 reaches 0.549, which is 2.6% higher than that of YOLOv13. The performance in terms of both localization accuracy and recognition accuracy is the best. Compared with RT-DETR, which is based on the transformer architecture, SOD-YOLO achieves significant superiority in terms of mAP@0.5 and mAP@0.5:0.95 while maintaining a similar recall rate, effectively overcoming the problem of insufficient accuracy of the model in the detection of small vehicle targets. Compared with each version of the YOLO series of algorithms, SOD-YOLO has achieved a comprehensive improvement in detection accuracy and localization capability and is more suitable for complex vehicle detection requirements in drone aerial photography scenarios.

To further verify the superiority and persuasiveness of our proposed SOD-YOLO, we conducted comparative experiments with several representative small-target detection algorithms for unmanned aerial vehicles. These include Gold-YOLO-N [42], DAMO-YOLO [43], and RTUAV-YOLO [20]. The detailed experimental results are presented in Table 9. From Table 9, it can be seen that DAMO-YOLO has the highest recall rate of 0.752 among all the algorithms, indicating its excellent performance in detecting difficult vehicle samples. RTUAV-YOLO and Gold-YOLO-N overall performance is inferior to our proposed method. This is mainly because these detectors perform poorly in detecting severely occluded or dense vehicle samples. Although the recall rate of our SOD-YOLO is slightly lower than DAMO-YOLO, its accuracy reaches 0.770, mAP@0.5 is 0.809, and mAP@0.5:0.95 is 0.549, demonstrating superior comprehensive detection performance for dense and mutually occluded vehicle targets in unmanned aerial vehicle aerial photography scenarios.

To verify the real-time detection performance of the proposed algorithm, we conducted relevant performance tests, and the results are shown in Table 10. The detection speed, GFlops and parameters of the proposed SOD-YOLO algorithm are 142 frames per second, 13.5 and 6,327,212, respectively. Compared with YOLOv13, the improved model is 136 frames per second slower in speed, and the GFlops and parameters increased by 7.3 and 387,8342, respectively. Thus, it can be seen that the improved model has a slightly reduced real-time performance, but it can still achieve real-time detection.

**Figure 11:**
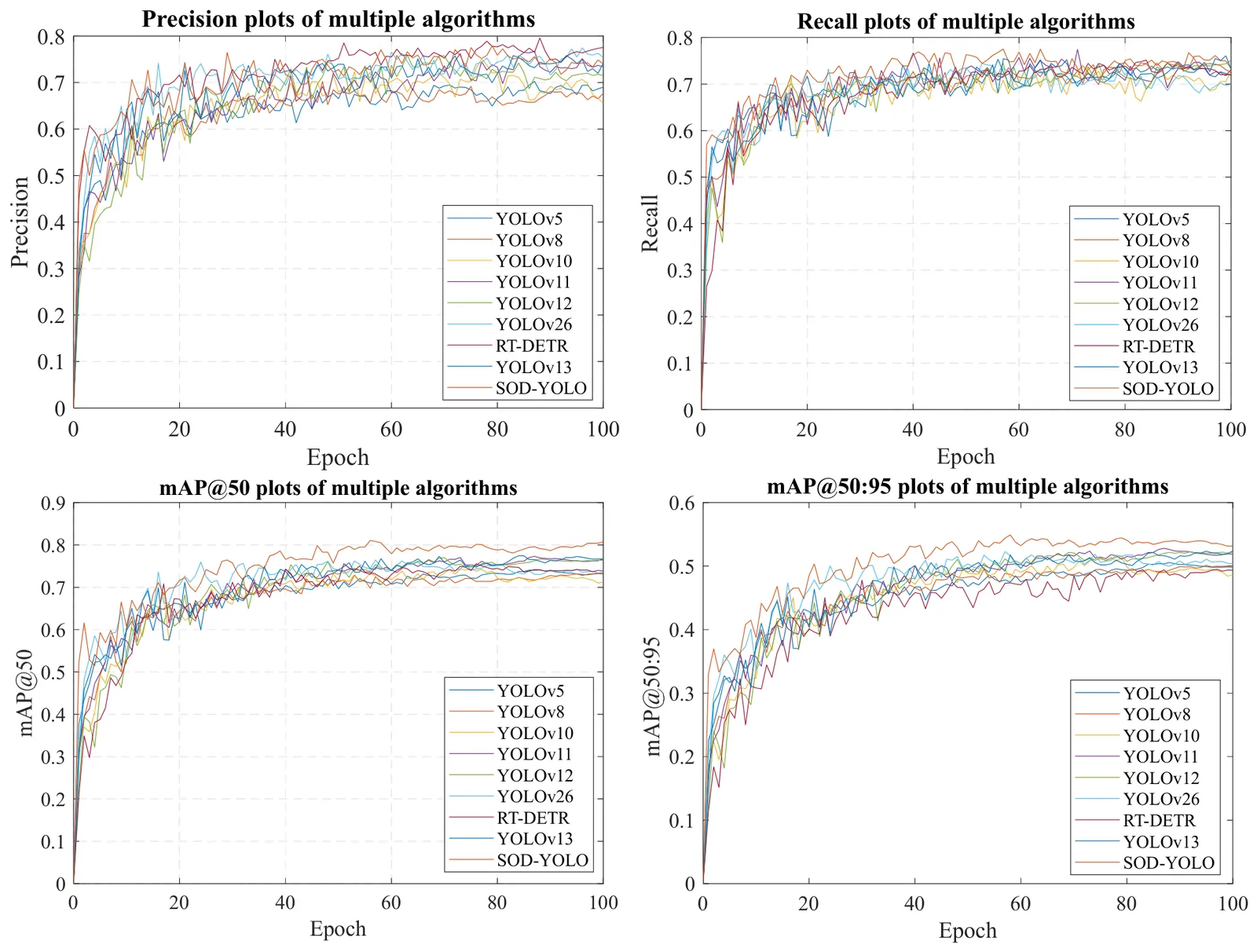
Performance curves of different comparison algorithms during the training process.

As shown in Figure 11, the precision, mAP@0.5 and mAP@0.5:0.95 curves of SOD-YOLO are at the highest level most of the time, and they converge more quickly. In the later training stage, they still maintain a stable upward trend and do not show obvious overfitting.

**Figure 12:**
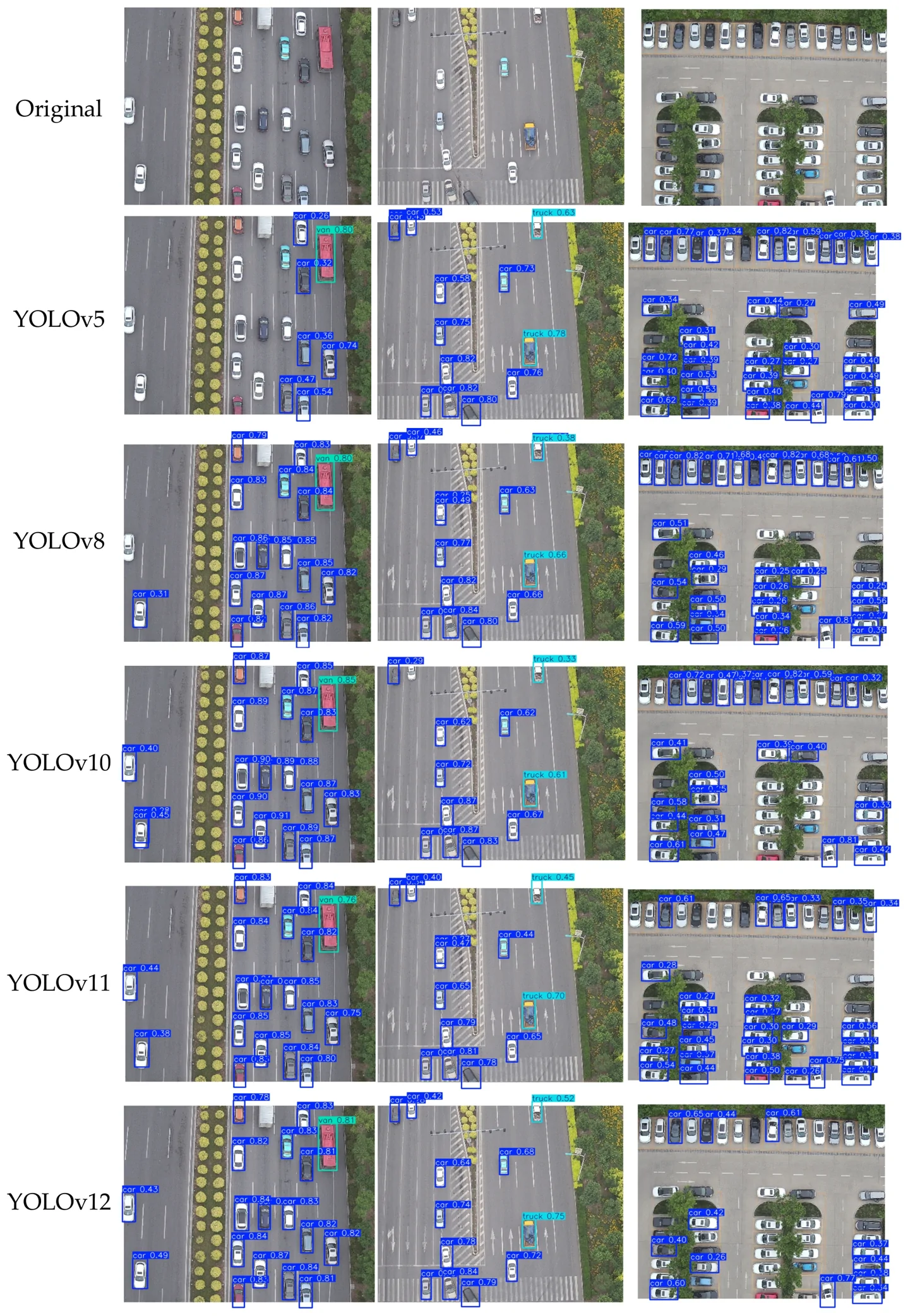

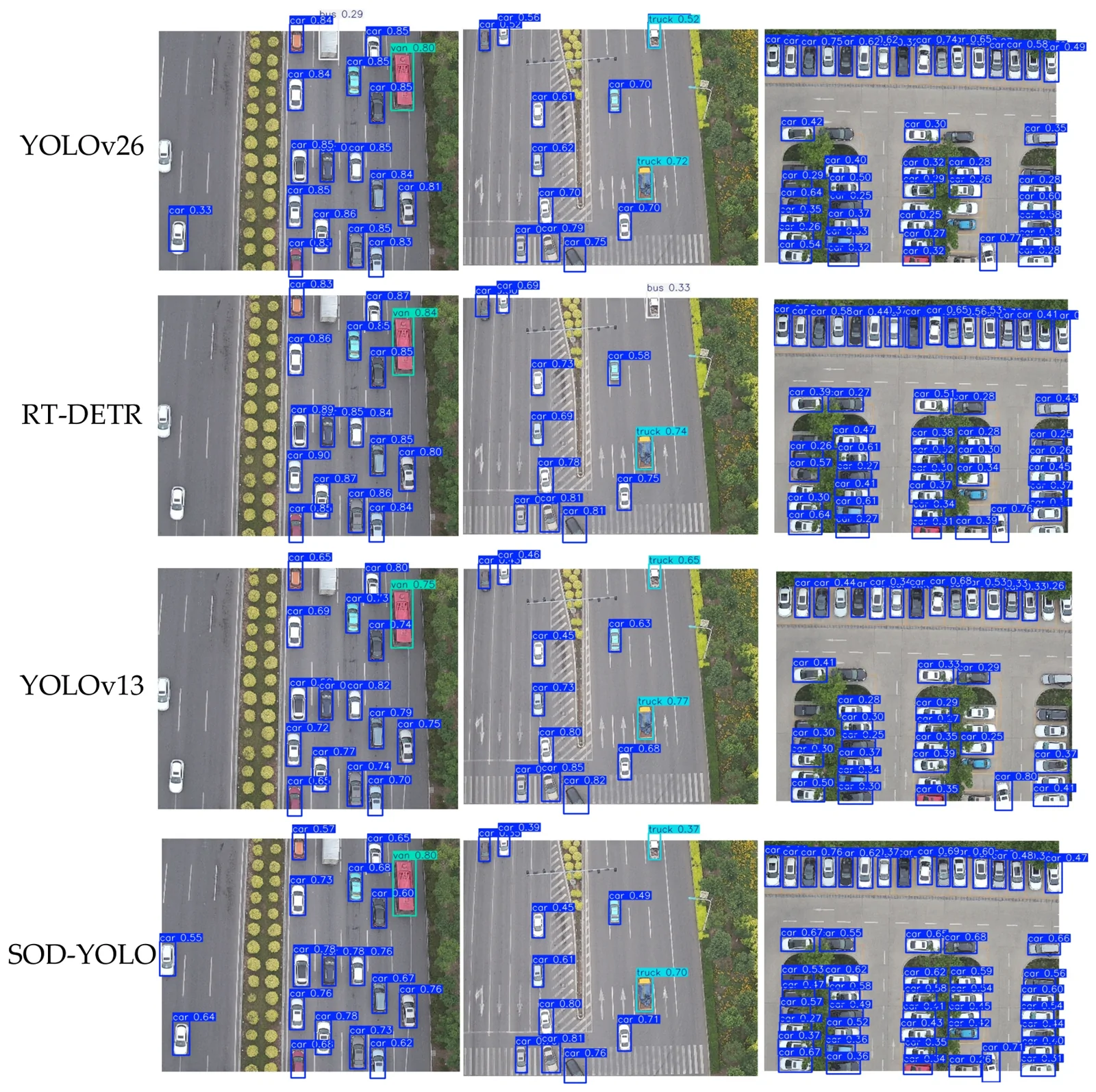
Qualitative detection comparison results between SOD-YOLO and other comparative algorithms on DroneVehicle dataset. Representative UAV aerial images containing dense parking lot vehicles, small-sized road vehicles and partially occluded vehicles are selected for comparison. The comparison intuitively demonstrates that SOD-YOLO obtains better detection performance for dense, small-scale and partially occluded vehicle targets.

In terms of the recall rate curve, SOD-YOLO is close to RT-DETR, and both are higher than the other YOLO series algorithms, further verifying its high recall ability for targets. The visual detection effect diagram shows that, compared with the other comparison algorithms, SOD-YOLO is better able to identify dense parking lots, small vehicles on the road, and vehicles that are partially obscured, as shown in Figure 12. This figure shows the qualitative detection comparison visualization results between SOD-YOLO and several comparative algorithms on the DroneVehicle dataset. The selected images cover typical UAV aerial scenarios such as dense parking lots, small-sized road vehicles and partially occluded vehicle targets. It can be intuitively observed that competing algorithms suffer from missed detections under dense arrangement and occlusion conditions. By contrast, SOD-YOLO achieves superior detection performance for these difficult samples, which verifies its effectiveness in handling dense, small-scale and partially occluded vehicle targets. The number of missed detections and false detections is significantly reduced, and the confidence of the detection boxes is higher. In complex backgrounds and occlusion scenarios, SOD-YOLO can accurately locate the vehicle edges and effectively distinguish the target from the interference area. This demonstrates stronger robustness and generalization ability.

**Figure 13:**
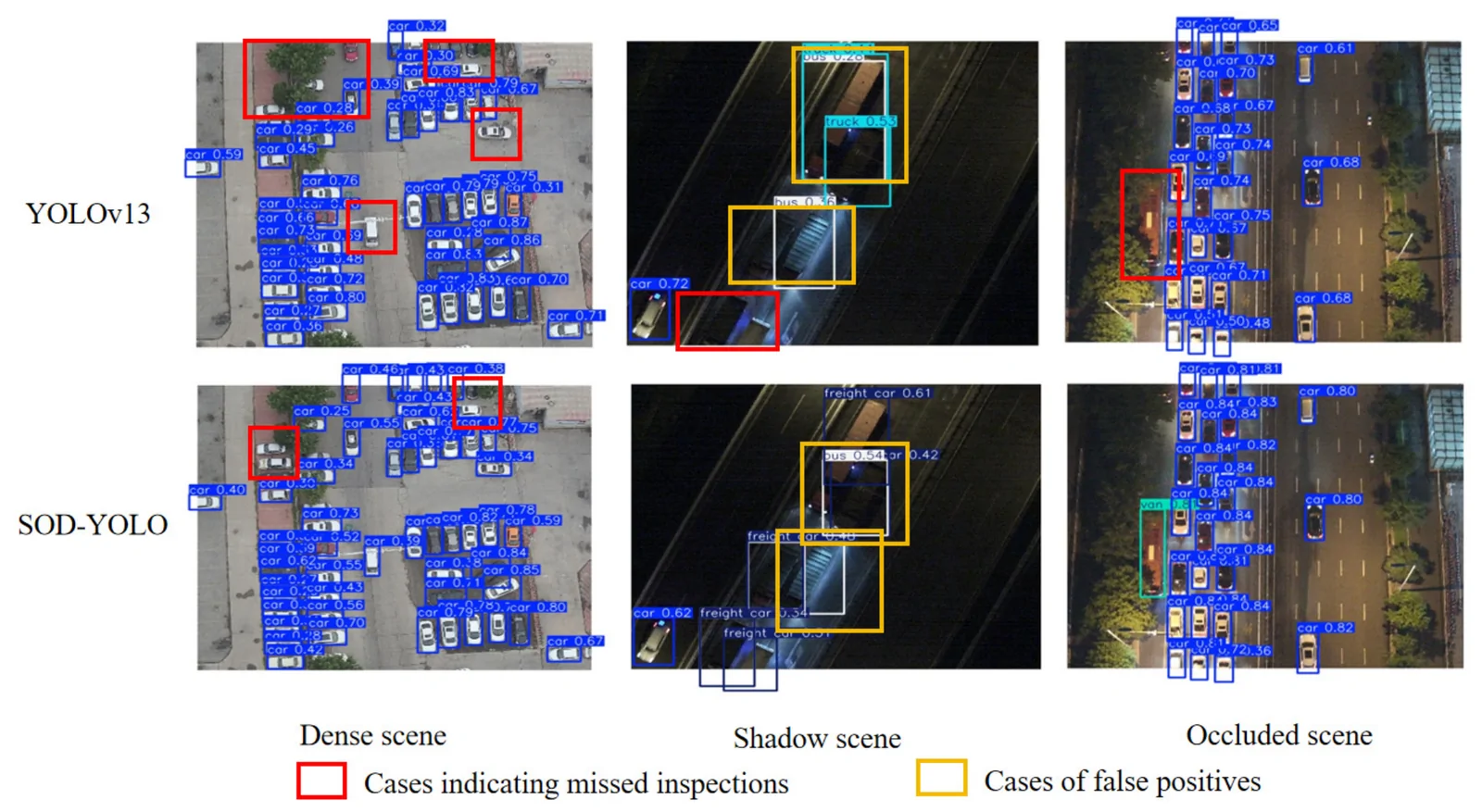
Visualization of the detection performance of the algorithm in various scenarios.

To make the algorithm more convincing, we selected representative aerial photography samples that included typical scenarios such as dense parking lots, shadow interference, and occlusion. Figure 13 presents the performance of the algorithm before and after the improvement in the detection output, as well as common false detections and missed detections and other failure situations.

The comparative experimental results fully demonstrate that our proposed SOD-YOLO algorithm effectively solves the problems of small-target feature loss, missed detection and false detection of occluded targets in unmanned aerial vehicle aerial scenes. While balancing the efficient inference speed of single-stage algorithms, a comprehensive improvement in detection accuracy and positioning capability is achieved.

To further investigate the cross-dataset generalization performance of the proposed SOD-YOLO, we conducted supplementary experiments on the VisDrone dataset [20]. The experimental results are summarized in Table 11. As can be observed from Table 11, SOD-YOLO achieves better precision, recall and mAP metrics compared with several representative baseline models. It obtains obvious performance gains for small aerial targets, which verifies that the improvements introduced in our model are not limited to the DroneVehicle dataset and can be generalized to other UAV-captured aerial object-detection scenarios.

**Figure 14:**
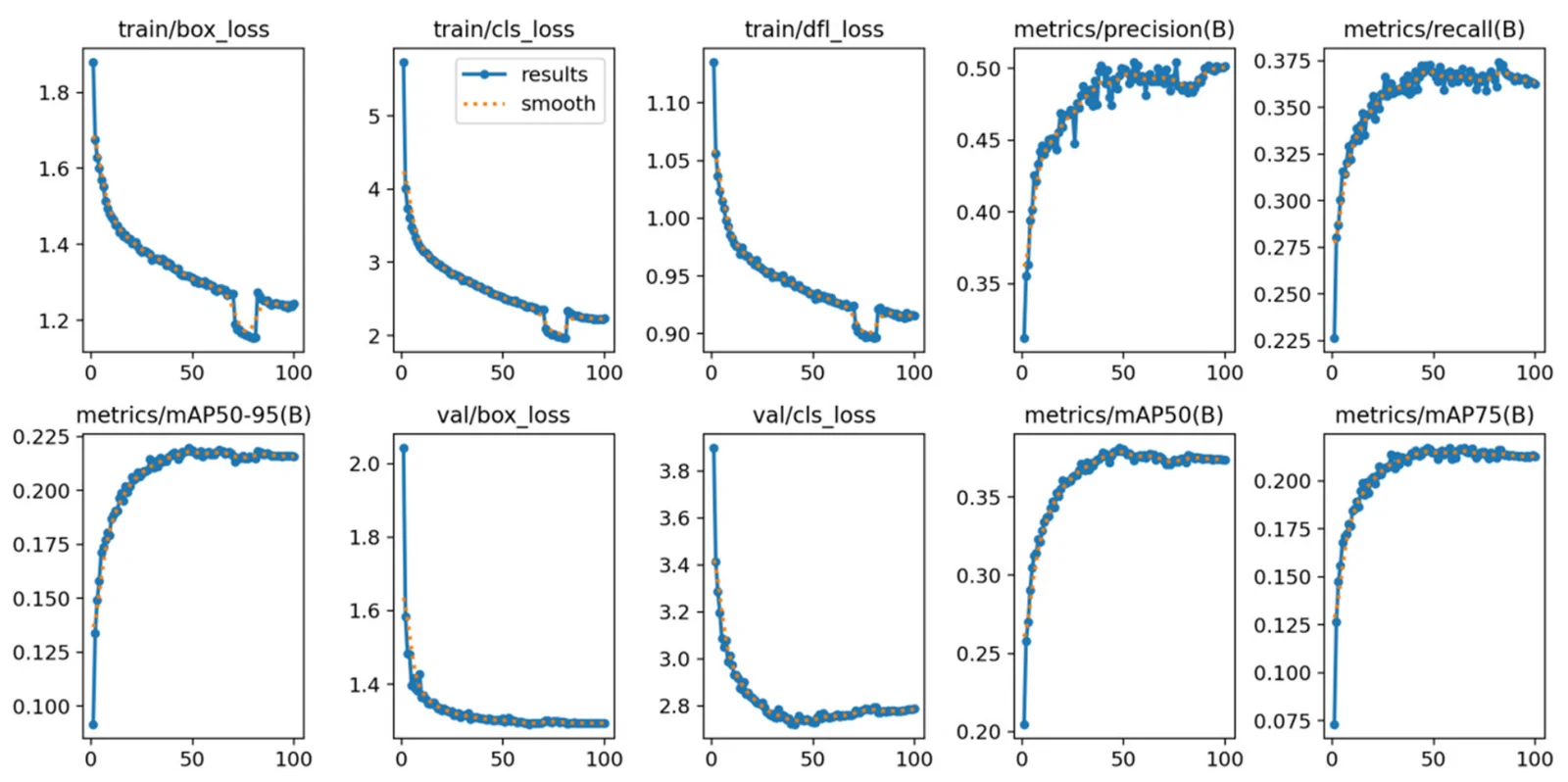
The performance curve of SOD-YOLO on the VisDrone dataset.

To observe the training convergence behavior of the proposed SOD-YOLO model, we plotted the training-process curves, as shown in Figure 14. It can be seen from the figure that both training loss and validation loss decrease rapidly in the early training phase and gradually become stable after about 60 epochs. Meanwhile, precision, recall and mAP metrics keep rising and finally reach a saturated state. There is no obvious divergence or severe over-fitting phenomenon between the training set and validation set, which demonstrates that SOD-YOLO converges steadily within 100 training epochs and obtains reliable detection performance.

## 4. Discussion

### 4.1. Computational Trade-Offs

As shown in Table 10, compared with YOLOv13, SOD-YOLO increases parameters from 2.45 M to 6.33 M and GFLOPs from 6.20 to 13.50, with desktop-GPU FPS dropping from 278 to 142. The extra computation is mainly caused by sliding-window self-attention in SOD-C2f. Despite higher overhead, SOD-YOLO achieves +3.6% mAP@0.5 and +2.6% mAP@0.5:0.95, and is far more lightweight than RT-DETR. There is a clear trade-off involved, which is to sacrifice some inference efficiency in order to achieve better detection results for small and obscured vehicles.

### 4.2. Applicability to Embedded UAV Hardware

All speed metrics are measured on desktop GPUs and cannot represent real onboard performance. Although lighter than transformer-based models, sliding-window self-attention involves frequent window operations which may bring latency on resource-limited UAV embedded chips. Direct real-time onboard deployment is not guaranteed. Further compression such as quantization or knowledge distillation will be required for practical airborne deployment, which will be studied in follow-up work.

### 4.3. Generalization

Our model is trained on the DroneVehicle dataset covering urban daytime and night UAV scenes. It works well for common urban vehicle targets, but lacks sufficient samples for heavy fog, heavy rain, rural scenarios and rare vehicle types. Generalization may degrade for unseen extreme scenes.

### 4.4. Failure-Case Analysis

Typical failure scenarios include the inability to detect very small vehicles due to the scarcity of pixels, resulting in incorrect identification. For vehicles that are severely obscured or only partially visible, there are misjudgments as negative cases. Occasionally, false alarms occur due to the similarity between the background texture and the vehicle. These are the main performance bottlenecks.

### 4.5. Limitations

The main limitations of SOD-YOLO are as follows. First, the increased computational overhead poses challenges for direct deployment on UAV embedded hardware without additional model compression. Second, its generalization performance is constrained by the diversity of training datasets. Third, the detection robustness for extremely small and heavily-occluded targets still requires further improvement.

### 4.6. Future Improvements

Future work will focus on adopting model compression techniques and conducting real embedded-hardware tests to realize UAV deployment, enriching datasets and applying adverse-weather augmentation to boost model generalization, exploring lightweight attention modules to improve feature extraction for hard samples with controlled computational cost, and introducing a tracking branch to support UAV vehicle tracking tasks.

## 5. Conclusions

To meet the challenges of a small target size, severe occlusion, and complex backgrounds in a low-altitude unmanned aerial vehicle (UAV) detection scenario, we propose the SOD-YOLO detection algorithm based on YOLOv13. On the preprocessed DroneVehicle dataset, this algorithm achieved an accuracy rate of 0.770, a recall rate of 0.748, an mAP@0.5 of 0.809, and an mAP@0.5:0.95 of 0.549. Compared with the baseline YOLOv13, mAP@0.5 and mAP@0.5:0.95 increased by 3.6% and 2.6% respectively.

This algorithm can effectively alleviate the problems of missed detection and false detection of small-sized and mutually occluded vehicle targets. It provides a feasible technical reference for intelligent traffic monitoring of low-altitude UAVs. However, the model still has certain limitations. Firstly, the computational cost increases, and it is difficult to directly deploy it on embedded hardware without compression. Secondly, the generalization ability is restricted by the diversity of the dataset. Finally, the detection robustness for extremely small targets and severely occluded targets still needs to be improved.

In subsequent research, we will conduct model compression and real-device testing for embedded hardware and expand the multi-scenario aerial photography dataset to improve the generalization ability. We will explore lightweight attention schemes for difficult samples and introduce a tracking branch to achieve the vehicle tracking task in UAV scenarios.

## Figures and Tables

**Table 1:** Evaluation results of baseline comparison. Each value is reported as mean ± standard deviation (std) over five independent training runs with different random seeds.

Model	Precision	Recall	mAP@0.5	mAP@0.5:0.95
YOLOv13	0.734 ± 0.002	0.737 ± 0.003	0.773 ± 0.003	0.523 ± 0.005
SOD-YOLO	0.770 ± 0.003	0.748 ± 0.003	0.809 ± 0.004	0.549 ± 0.005

**Table 2:** Evaluation results of ablation experiments. (√ denotes the inclusion of the corresponding module).

YOLOv13	SOD-C2f	CA	SPDConv	Precision	Recall	mAP@0.5	mAP@0.5:0.95
√				0.734	0.737	0.773	0.523
√	√			0.744	0.752	0.762	0.526
√		√		0.737	0.754	0.783	0.537
√			√	0.764	0.731	0.788	0.541
√	√	√		0.767	**0.775**	0.786	**0.55**
√	√	√	√	**0.77**	0.748	**0.809**	0.549

Note: The bolded items in the table indicate the best effects.

**Table 3:** Ablation results of different sliding window sizes for SWSA on a DroneVehicle dataset.

Window Size	Precision	Recall	mAP@0.5	mAP@0.5:0.95
4 × 4	0.761	0.739	0.784	0.535
8 × 8	0.77	0.748	0.809	0.549
16 × 16	0.768	0.744	0.802	0.544

**Table 4:** Ablation results of different attention-head numbers for SWSA.

Attention Module	Precision	Recall	mAP@0.5	mAP@0.5:0.95
2	0.754	0.731	0.791	0.528
4 (Ours)	0.77	0.748	0.809	0.549
8	0.783	0.745	0.812	0.547

**Table 5:** Ablation results of different SPDConv positions.

SPDConv Positions	Precision	Recall	mAP@0.5	mAP@0.5:0.95
a: Before CA (Ours)	0.77	0.748	0.809	0.549
b: After CA	0.758	0.726	0.778	0.526

**Table 6:** Ablation results of different CA placement.

CA Placement	Precision	Recall	mAP@0.5	mAP@0.5:0.95
a: Before Concat (Ours)	0.77	0.748	0.809	0.549
b: After Concat	0.62	0.73	0.651	0.433

**Table 7:** Ablation results of relative positional encoding.

Relative Positional Encoding	Precision	Recall	mAP@0.5	mAP@0.5:0.95
no	0.495	0.512	0.557	0.386
yes (Ours)	0.77	0.748	0.809	0.549

**Table 8:** Comparison of vehicle target detection algorithms.

Method	Precision	Recall	mAP@0.5	mAP@0.5:0.95
YOLOv5	0.682	0.725	0.741	0.506
YOLOv8	0.692	0.728	0.743	0.511
YOLOv10	0.746	0.703	0.742	0.514
YOLOv11	0.738	0.741	0.772	0.529
YOLOv12	0.713	0.729	0.763	0.522
YOLOv26	0.749	0.722	0.765	0.523
RT-DETR	0.769	**0.753**	0.751	0.502
YOLOv13	0.734	0.737	0.773	0.523
**SOD-YOLO**	**0.77**	0.748	**0.809**	**0.549**

Note: The bolded items in the table indicate the best effects.

**Table 9:** Comparison with recent UAV-specific detectors on the DroneVehicle dataset.

Method	Precision	Recall	mAP@0.5	mAP@0.5:0.95
Gold-YOLO-N	0.701	0.722	0.742	0.519
DAMO-YOLO	0.738	0.752	0.781	0.527
RTUAV-YOLO	0.726	0.738	0.766	0.522
YOLOv13	0.734	0.737	0.773	0.523
SOD-YOLO	0.77	0.748	0.809	0.549

**Table 10:** Real-time performance indicators of the model.

Model	ms/Image	FPS	GFlops	Parameters
YOLOv8	10.3	97	8.20	3,011,823
YOLOv11	13.2	76	6.44	2,590,815
RTDETR	6.90	145	103.5	31,994,015
YOLOv13	3.60	278	6.2	2,448,870
SOD-YOLO	7.04	142	13.5	6,327,212

**Table 11:** Real-time performance indicators of the model on the VisDrone dataset.

Method	Precision	Recall	mAP@0.5	mAP@0.5:0.95
YOLOv8	42.5	33.9	33.3	19.6
YOLOv11	42.3	33.6	33.1	19.3
RTUAV-YOLO	44.7	35.6	35.9	21.3
YOLOv13	39.6	29.3	28.8	15.9
SOD-YOLO	49.5	37.3	38.1	22

## Data Availability

The data presented in this study are available on request from the corresponding author.
